# The c.*52 *A/G* and c.*773 *A/G* Genetic Variants in the UTR′3 of the *LDLR* Gene Are Associated with the Risk of Acute Coronary Syndrome and Lower Plasma HDL-Cholesterol Concentration

**DOI:** 10.3390/biom10101381

**Published:** 2020-09-29

**Authors:** Gilberto Vargas-Alarcon, Oscar Perez-Mendez, Julian Ramirez-Bello, Rosalinda Posadas-Sanchez, Hector Gonzalez-Pacheco, Galileo Escobedo, Betzabe Nieto-Lima, Elizabeth Carreon-Torres, Jose Manuel Fragoso

**Affiliations:** 1 Department of Molecular Biology, Instituto Nacional de Cardiología Ignacio Chavez, Mexico City 14080, Mexico; gvargas63@yahoo.com (G.V.-A.); opmendez@yahoo.com (O.P.-M.); betsy@ciencias.unam.mx (B.N.-L.); qfbelizabethcm@yahoo.es (E.C.-T.); 2 School of Engineering and Scienses, Tecnologico de Monterrey, Campus Ciudad de Mexico, Mexico City 14380, Mexico; 3 Research Unit on Endocrine and Metabolic Diseases, Hospital Juarez de México, Mexico City 01460, Mexico; dr.julian.ramirez.hjm@gmail.com; 4 Department of Endocrinology, Instituto Nacional de Cardiología Ignacio Chavez, Mexico City 14080, Mexico; rossy_posadas_s@yahoo.it; 5 Unit Coronary, Instituto Nacional de Cardiología Ignacio Chavez, Mexico City 14080, Mexico; hectorglezp@hotmail.com; 6 Unit of the Experimental Medicine, Hospital General de Mexico, Dr. Eduardo Liceaga, Mexico City 06726, Mexico; gescobedog@msn.com

**Keywords:** genetics, single nucleotide polymorphism, acute coronary syndrome

## Abstract

Dyslipidemia has a substantial role in the development of acute coronary syndrome (ACS). Low-density lipoprotein receptor (LDLR) plays a critical role in plasma lipoprotein hemostasis, which is involved in the formation of atherosclerotic plaque. This study aimed to evaluate whether *LDLR* gene polymorphisms are significantly associated with ACS and the plasma lipids profile. Three *LDLR* gene polymorphisms located in the *UTR′3* region (c.*52 *A/G*, c.*504 *A/G*, and c.* 773 *A/G*) were determined using TaqMan genotyping assays in a group of 618 ACS patients and 666 healthy controls. Plasma lipids profile concentrations were determined by enzymatic/colorimetric assays. Under co-dominant and recessive models, the c.*52 A allele of the c.*52 A/G polymorphism was associated with a higher risk of ACS (OR = 2.02, *pC*_Co-dom_ = 0.033, and OR = 2.00, *pC*_Res_ = 0.009, respectively). In the same way, under co-dominant and recessive models, the c.*773 G allele of the c.*773 A/G polymorphism was associated with a high risk of ACS (OR = 2.04, *pC*_Co-dom_ = 0.027, and OR = 2.01, *pC*_Res_ = 0.007, respectively). The “*AAG”* haplotype was associated with a high risk of ACS (OR = 1.22, *pC* = 0.016). The c.*52 AA genotype showed a lower HDL-C concentration than individuals with the *GG* genotype. In addition, carriers of c.*773 GG genotype carriers had a lower concentration of the high-density lipoprotein-cholesterol (HDL-C) than subjects with the *AA* genotype. Our data suggest the association of the *LDLR* c.*773 A/G and LDLR c.*52 A/G polymorphisms with both the risk of developing ACS and with a lower concentration of HDL-C in the study population.

## 1. Introduction

Acute coronary syndrome (ACS) constitutes a worldwide public health problem. It is a complex disease resulting from the interaction of genetic and environmental factors, as well as traditional cardiovascular risk factors [1,2]. This syndrome is a consequence of atherosclerosis by the excessive accumulation of cholesterol, which results in the formation of the atherosclerotic plaque associated with a strong inflammatory component [1,2,3]. The low-density lipoprotein receptor (LDLR) is a cell membrane glycoprotein that functions in the binding and internalization of circulating cholesterol-containing lipoprotein particles. The LDL receptor is ubiquitously expressed and is a key receptor for maintaining cholesterol homeostasis in humans [3,4,5]. This receptor mediates endocytosis of plasma lipoproteins containing apolipoprotein B, as well as remnants of triglyceride-rich lipoprotein metabolism, which are the precursors of plasma low-density lipoprotein cholesterol (LDL-C), which plays an important role in the atherosclerotic plaque [4,5,6].

Previous reports have shown a large number of the genetic variants in the *LDLR* gene that play an important role in the development of hypercholesterolemia and cardiovascular diseases in different populations; however, less than 15% have functional evidence [7,8]. Nonetheless, in recent years, three novel single nucleotide polymorphisms [*LDLR* UTR′3 c.*52 *A/G* (rs14158), *LDLR* UTR′3 c.*504 *A/G* (rs2738465), and *LDLR* UTR′3 c.* 773 *A/G* (rs2738466)] in the 3′ untranslated region (UTR′3) of the *LDLR* gene (located in p13.1-13.3 of the chromosome 19) have been associated with higher levels of LDL-C and a greater risk of the developing hypercholesterolemia, the principal cardiovascular risk factor in atherosclerosis [9,10,11].

In this context, considering the important role of the LDL receptor in the uptake of low-density lipoprotein cholesterol (LDL-C) associated with the atherosclerotic plaque formation, the present study aimed to establish the role of the *LDLR* c.*52 *A/G*, *LDLR* c.*504 *A/G*, and *LDLR* c.* 773 *A/G* polymorphisms in the susceptibility to develop ACS. Furthermore, we evaluated whether these polymorphisms are associated with lipid profile plasma concentrations in a Mexican population sample.

## 2. Materials and Methods

### 2.1. Characteristics of the Study Population

We used the sample size calculation for unmatched cases and controls study with a power of 80% and an alpha error of 0.05 [12]. The study included 618 patients with ACS and 666 healthy controls unmatched by age or gender. From July 2010 to July 2015, 618 patients with ACS (82% men and 18% women, with a mean age of 58 ± 10.5 years) were referred to the Instituto Nacional de Cardiologia Ignacio Chavez. The patient inclusion criterion was the diagnosis of ACS; this disease was identified and classified by either an ST-elevation myocardial infarction (STEMI) or a non-ST-elevation ACS (NSTE-ACS) based on clinical characteristics, electrocardiographic changes, and biochemical markers of cardiac necrosis (creatinine kinase isoenzymes, creatinine phosphokinase, or troponin I above the upper limit of normal). The European Society of Cardiology (ESC) and American College of Cardiology (ACC) definitions were followed [13,14]. The diagnosis of NSTE-ACS included non-STEMI and unstable angina. The diagnosis of non-STEMI was angina or discomfort at rest with ST-segment changes on ECG indicating ischemia [ST-segment depression or transient elevation (≥1 mm) in at least two contiguous leads and/or prominent T-wave inversion] with a positive biomarker indicating myocardial necrosis. Patients with clinical features and/or electrocardiographic expression of non-STEMI (albeit with normal cardiac biomarker levels) were diagnosed with unstable angina [13,14]. Moreover, 666 healthy controls were included (68% men and 32% women with a mean age of 54 ± 7.65 years) from the cohort of the Genetics of Atherosclerotic Disease (GEA) Mexican study. The GEA study investigates the genetic factors associated with premature coronary artery disease (CAD), atherosclerosis, and other coronary risk factors in the Mexican population [15]. All subjects were asymptomatic and healthy individuals without a family history of premature CAD or atherosclerosis; they were recruited from June 2009 to June 2013 from blood bank donors and with the assistance of brochures posted in social service centers. The exclusion criteria included the use of anti-dyslipidemic and anti-hypertensive drugs at the time of the study, congestive heart failure, and liver, renal, thyroid, or oncological disease. Additionally, the control subjects had a zero coronary calcium score determined by computed tomography, indicating the absence of subclinical atherosclerosis [15]. To assess the contributions of the *LDLR* UTR′3 c.*52 *A/G*, *LDLR* UTR′3 c.*504 *A/G*, and *LDLR* UTR′3 c.* 773 *A/G* SNPs genotypes on the plasma lipids levels, we selected only the healthy controls group. All the included subjects were ethnically matched and considered Mexican mestizos only if they and their ancestors (at last three generations) had been born in the country. The study complies with the Declaration of Helsinki and was approved by the Ethics and Research commission of Instituto Nacional de Cardiologia Ignacio Chavez. Written informed consent was obtained from all individuals enrolled in the study.

### 2.2. Laboratory Analyses

Cholesterol and triglycerides plasma concentrations were determined by enzymatic/colorimetric assays (Randox Laboratories, Crumlin, Country Antrim, UK). HDL-cholesterol (C) concentrations were determined after precipitation of the apo B-containing lipoproteins by the method of the phosphotungstic acid-Mg^2+^. The LDL-C concentration was determined in samples with a triglyceride level lower than 400 mg/dL with the Friedewald formula [16]. Dyslipidemia was defined as the presence of one or more of the following conditions: cholesterol > 200 mg/dL, LDL-C > 130 mg/dL, HDL-C < 40 mg/dL, or triglycerides > 150 mg/dL, according to the guidelines of the National Cholesterol Education Project (NCEP) Adult Treatment Panel (ATP III) [17]. Type 2 diabetes mellitus (T2DM) was defined with a fasting glucose ≥ 126 mg/dL; it was also considered when participants reported glucose-lowering treatment or a physician diagnosis of T2DM. Hypertension was defined by a systolic blood pressure ≥ 140 mmHg, diastolic blood pressure ≥ 90 mmHg, or the use of oral antihypertensive therapy [15].

### 2.3. Genetic Analysis

DNA extraction was performed from peripheral blood in agreement with the method described by Lahiri and Nurnberger [18]. The *LDLR* UTR′3 c.*52 *A/G* (rs14158), *LDLR* UTR′3 c.*504 *A/G* (rs2738465), and *LDLR* UTR′3 c.* 773 *A/G* (rs2738466) SNPs were genotyped using 5′ exonuclease TaqMan genotyping assays on a 7900HT Fast Real-Time PCR System according to manufacturer’s instructions (Applied Biosystems, Foster City, CA, USA). To avoid genotyping errors, 10% of the samples were assayed in duplicate; the results were concordant for all cases.

### 2.4. Inheritance Models Analysis

The association of the c.*52 *A/G*, c.*504 *A/G*, and c.* 773 *A/G* SNPs with ACS patients was perform under the following inheritance model: additive (major allele homozygotes versus heterozygotes versus minor allele homozygotes), codominant (major allele homozygotes versus minor allele homozygotes), dominant (major allele homozygotes versus heterozygotes + minor allele homozygotes), over-dominant (heterozygotes versus major allele homozygotes + minor allele homozygotes), and recessive (major allele homozygotes + heterozygotes versus minor allele homozygotes) using logistic regression, adjusting for cardiovascular risk factors.

### 2.5. Analysis of the Haplotypes

The linkage disequilibrium analysis (LD, D″) and haplotypes construction were performed using Haploview version 4.1 (Broad Institute of Massachusetts Institute of Technology and Harvard University, Cambridge, MA, USA).

### 2.6. Functional Prediction Analysis

Two in silico programs, the ESEfinder 3.0 and SNP Function Prediction, were used to predict the possible functional effect of the LDLR SNPs. Both web-based tools (ESEfinder2.0 and SNPinfo) analyze the localization of the SNPs (e.g., 5′-upstream, 3′-untranslated regions, intronic) and their possible functional effects, such as amino acid changes in protein structure, transcription factor binding sites in the promoter or intronic enhancer regions, and alternative splicing regulation by disrupting exonic splicing enhancers (ESE) or silencers [19,20].

### 2.7. Statistical Analysis

All statistical analyses in this study were performed using SPSS version 18.0 (SPSS, Chicago, IL, USA). The Mann-Whitney *U* test was used to compare the continuous variables (e.g., age, body mass index (BMI), blood pressure, glucose, total cholesterol, HDL-C, LDL-C, and triglycerides) between control and ACS groups. For the categorical variables (e.g., gender, hypertension, T2DM, dyslipidemia, and smoking habit), chi-squared or Fisher’s exact tests were performed. All *p*-values were corrected (pC) by the Bonferroni test. The values of pC < 0.05 were considered statistically significant, and all odds ratios (OR) were presented with 95% confidence intervals. The occurrence of the ACS in our study was based in the OR values: (a) OR = 1 does not affect the odds of developing ACS, (b) OR > 1 is associated with higher odds of developing ACS, and (c) OR < 1 is associated with lower odds of developing ACS. To evaluate Hardy-Weinberg equilibrium (HWE), we used the chi-squared test. The Mann-Whitney U test was used for establishing the contributions of the genotypes on the lipid plasma levels. Values were expressed as means ± SD, and statistical significance was set at *p* < 0.05. The statistical power to detect an association with ACS was 0.80 according to the QUANTO software [21].

## 3. Results

### 3.1. Characteristics of the Study Population

Anthropometrics and biochemical parameters of the ACS patients and healthy controls are presented in Table 1. Patients with ACS have higher levels of blood pressure, glucose, and higher prevalence of hypertension, diabetes, and dyslipidemias than control subjects. On the other hand, ACS patients presented lower levels of total cholesterol, LDL-C, and triglycerides than healthy controls. This phenomenon could be due to the treatment with statins received by the group of patients.

### 3.2. Allele and Genotype Frequencies

Genotype frequencies in the polymorphic sites were in HWE. The allele and genotype frequencies of the *LDLR* SNPs in ACS patients and healthy controls are shown in Table 2. The frequencies allelic of the c.*52 *A/G,* c.*504 A/G, and c.*773 *A/G* SNPs located in the *LDLR* gene showed that the c.*52 *A*, c.*504 A, and c.*773 *G* alleles were associated with the risk of developing ACS (OR = 1.20, *pC* = 0.02, OR = 1.18, *pC* = 0.02, and OR = 1.22, *pC* = 0.01 respectively) (Table 2). In addition, we corroborated the association according to the inheritance models. In this context, the association of the c.*504 A/G polymorphism loses significance statistically (pC > 0.05). Nonetheless, the c.*52 *A/G* and c.*773 *A/G* polymorphisms were associated with the presence of ACS (Table 2). Under co-dominant and recessive models, the c.*52 *AA* genotype of the c.*52 *A/G* polymorphism was associated with a greater risk of ACS (OR = 2.02, *pC*_Co-dom_ = 0.033, and OR = 2.00, *pC*_Res_ = 0.009, respectively). In the same way, under co-dominant and recessive models, the c.*773 *G* genotype of the c.*773 *A/G* polymorphism was associated with a high risk of ACS (OR = 2.04, *pC*_Co-dom_ = 0.027, and OR = 2.01, *pC*_Res_ = 0.007, respectively). All models were adjusted for gender, age, blood pressure, BMI, glucose, total cholesterol, HDL-C, LDL-C, triglycerides, and smoking habits.

### 3.3. Linkage Disequilibrium Analysis

The linkage disequilibrium analysis between the c.*52 *A/G*, c.*504 *A/G*, and c.* 773 *A/G* SNPs located in the *LDLR* gene showed three common haplotypes (Table 3). Two of them showed significant differences between patients with ACS and healthy controls. The “*GGA*” haplotype was associated with a low risk of developing ACS (OR = 0.84, 95% CI: 0.71–0.99, *pC* = 0.023), whereas the “*AAG*” haplotype was associated with high risk of developing the same syndrome (OR = 1.22, 95% CI: 1.02–1.46, *pC* = 0.016). In this study, we did not find any other haplotype because these SNPs are in almost complete linkage disequilibrium (D′ ≈ 1), which results in the joint co-segregation of these polymorphisms in the cases and controls (data not shown).

### 3.4. Functional Prediction

According, with the in silico programs ESEfinder 3.0 and SNP Function Prediction [19,20], the functional prediction analysis showed that the presence of the *A* allele of the c.*504 *A/G* polymorphism potentially produced a binding motif for the miR-200a microRNA. Moreover, a binding motif for miR-638 is predicted for the *G* allele of the c.* 773 *A/G* SNP. This analysis suggest that these polymorphisms located in the UTR′3 of the LDLR gene could be influence splicing or mRNA stability altering expression levels.

### 3.5. Association of Polymorphisms and Haplotypes with Plasma Lipids Levels

**Figure 1:**
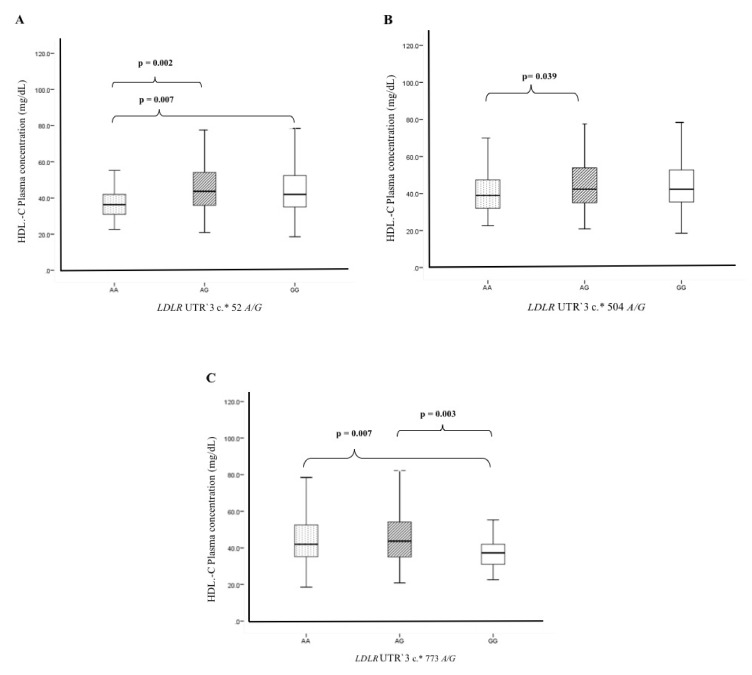
Genetic contribution of the *LDLR* UTR′3 c.*52 A/G, *LDLR* UTR′3 c.*504 *A/G*, and *LDLR* UTR‘3 c.* 773 *A/G* polymorphisms on HDL-C levels. (**A**) The *AA* genotype of the *LDLR* UTR′3 c.*52 *A/G* SNP showed low HDL-C levels in plasma when compared to *AG/GG* genotypes. (**B**) The *AA* genotype of the *LDLR* UTR′3 c.*504 *A/G* polymorphism showed lower HDL-C levels in plasma than the *AG* genotype. (**C**) The *GG* genotype of the *LDLR* UTR′3 c.* 773 *A/G* showed low HDL-C levels in plasma when compared to *AA/AG* genotypes.

**Figure 2:**
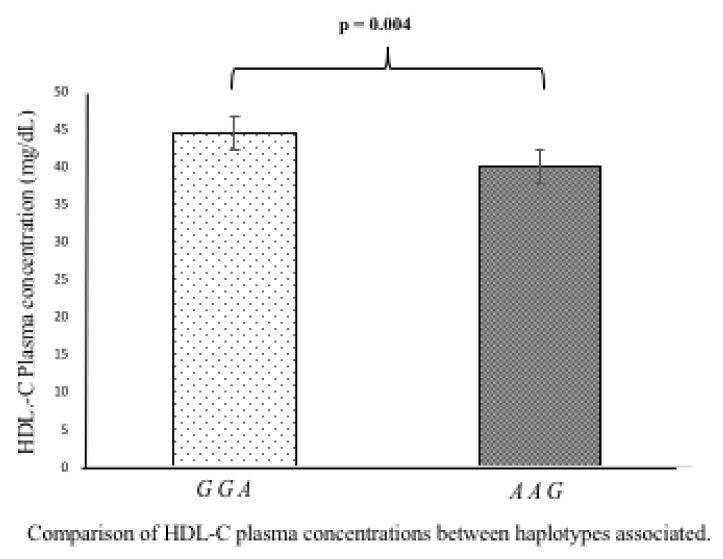
Contribution of the “*GGA*” and “*AAG*” on HDL-C levels. The “*AAG*” haplotype risk showed a lower concentration of HDL-C in plasma when compared to “*GGA*” haplotype of low risk (*p* = 0.004).

To define the possible functional effect of c.*52 *A/G*, c.*504 *A/G*, and c.* 773 *A/G* SNPs, we determined the plasma lipids levels (total cholesterol, LDL-C, HDL-C, and triglycerides), as well as the risk cardiovascular factors (BMI, blood pressure, glucose) in individuals with different genotypes of these three polymorphisms. For this analysis, we selected only the healthy controls group. We did not include the plasma-lipid level analysis in patients with ACS because, in the setting of the coronary syndrome, these levels may be altered using anti-dyslipidemic or anti-hypertensive drugs [8,9,10]. The analysis showed that the c.*52 *A/G*, c.*504 *A/G*, and c.* 773 *A/G* SNPs were not associated with the following parameters: total cholesterol, LDL-C, triglycerides, BMI, blood pressure, and glucose (Supplementary Table S1). However, we observed significant differences in HDL-C plasma levels when subjects were grouped by these SNPs. As for the c.*52 *A/G* SNP, individuals with the *AA* genotype showed a lower concentration of HDL-C in plasma (38 ± 10.4 mg/dL) than individuals with either *AG* (45.5 ± 13.7 mg/dL, *p* = 0.002) or *GG* genotypes (44.3 ± 13.2 mg/dL, *p* = 0.007) (Figure 1A). Alternatively, subjects carrying c.*773 *GG* genotype had a lower HDL-C plasma concentration (38 ± 10.3 mg/dL) than carriers of either the *AA* (44.4 ± 13.1 mg/dL, *p* = 0.007) or *AG* genotype (45.4 ± 14.9 mg/dL, *p* = 0.003) (Figure 1C). Although the c.*504 *A/G* polymorphism was not associated with ACS development, individuals with the c.*504 *AA* genotype had a lower HDL-C plasma concentration than individuals with the *AG* genotype (41.7 ± 13.8 mg/dL, *p* = 0.039) (Figure 1B). In addition, the analysis of the haplotypes (*GGA* and *AAG*) showed significant differences when compared with HDL-C plasma concentrations. The “*AAG*” haplotype risk showed a lower concentration of HDL-C in plasma (39.9 ± 12.3 mg/dL) when compared to “*GGA*” haplotype of low risk (44.5 ± 13.24, *p* = 0.004) (Figure 2).

## 4. Discussion

ACS is a multifactorial and polygenic disorder consequence of atherosclerosis, in which the excessive accumulation of cholesterol plays an important role. In the present study, we focused on the LDL receptor, which is a cell membrane glycoprotein that functions in the binding and internalization of circulating cholesterol-containing lipoprotein particles. The LDL receptor is ubiquitously expressed and is a key receptor for maintaining cholesterol homeostasis in humans [3,4,5]. We studied three polymorphisms (c.*52 *A/G*, c.*504 *A/G*, and c.*773 *A/G)* located in the 3′ untranslated region of the *LDLR* gene in ACS patients and healthy controls. According to our analysis, the c.*52 *A/G* and c.*773 *A/G* SNPs were associated with the risk of developing ACS, as well as with lower plasma HDL-C concentrations. To the best of our knowledge, this study is the first to describe the association between these polymorphisms and the presence of ACS. In this context, the association of these SNPs with several diseases in different populations is scarce and controversial. For example, in agreement with our data, van Zyl et al. reported that the *A* allele of the c.*52 *A/G* SNP increased the risk of developing familial hypercholesterolemia in the African population [11]. In the same way, De Castro-Oros et al. reported that the hypercholesterolemic subjects with the c.*52 *A,* c.*504 *A,* and c.*773 *G* alleles have a lower response to the anti-dyslipidemic drug Armolipid Plus; in their study, the authors suggested that these SNPs increased the risk of developing hypercholesterolemia in a Spanish population [10]. In contrast, Zambrano et al. reported that the *A* allele of the c.*52 *A/G* SNP decreased the risk of the developing hypercholesterolemia in a Brazilian population [9]. By the same token, Chen et al. reported that c.*52 *A/G*, c.*504 *A/G,* and c.*773 *A/G* polymorphisms were not associated with the risk of developing coronary heart diseases in a Chinese population [22]. Although in our study the c.*504 A/G polymorphism showed a moderate association with the risk of developing ACS (*p*C = 0.06), van Zyl et al. reported that this polymorphism increased the risk of developing familial hypercholesterolemia in an African population (*p* = 0.051) [11]. In addition, we found that the “AAG” haplotype was associated with a high risk of developing ACS and with lower plasma HDL-C levels, whereas the “GGA” haplotype was associated with a low risk and higher plasma HDL-C levels.

According to data in the literature, the impact of the *LDLR* gene polymorphisms on lipid plasma concentration has been proposed as the mechanism that explains the relationship between these SNPs and the higher risk of developing familial hypercholesterolemia [7,8]. In this context, recent studies have associated the *LDLR* polymorphisms (c.*52 *A/G*, c.*504 *A/G*, and c.*773 *A/G*) with low levels of plasma lipids and the risk of developing familial hypercholesterolemia [9,10,11,23]. For example, Li et al. reported that c.*773 *G/G* genotype is associated with decreased plasma levels of HDL-C in healthy individuals of China [23]. In the same way, van Zyl et al. documented that c.*52 *A/A* and c.*504 *A/A* genotypes are associated with increased levels of LDL-C in the healthy black South African population [11]. Our results showed that c.*52 *A/A*, c.*504 *A/A*, and c.*773 *G/G* genotypes were associated with low HDL-C levels. In contrast, Chen et al., studying a Chinese population, reported that these polymorphisms were not associated with altered plasma lipid levels in patients with coronary heart diseases and healthy controls [22]. As far as we know, the precise mechanism by which low HDL-C levels are associated with hypercholesterolemia and adverse events, such as ACS, remains to be elucidated. Nonetheless, data in the literature provide evidence that the reduction of plasma HDL-C is due to the defective assembly of nascent HDL by hepatocyte Abca1 (ATP binding cassette transport A1) and increased plasma clearance of HDL protein and cholesteryl ester [24,25]. Moreover, experimental studies in mice have shown that (i) the hepatic LDL receptor stimulated plasma HDL selective cholesteryl ester uptake, and (ii) sterol trafficking into reverse cholesterol transport decreased HDL-C levels, when hepatocyte Abca1 was deficient [24,25,26]. Additionally, using bioinformatics tools, we determined the potential effect of the *LDLR* gene polymorphisms; no evidence of a functional motif was found for the c.*52 *A/G* polymorphism. Nonetheless, the analysis of the c.*504 *A/G* polymorphism showed that *A* allele produced a binding motif for miR-200a; this microRNA regulates the kelch-like EHC-associated protein 1 (Keap1)/nuclear factor erythroid 2-related factor (Nrf2) signaling axis, which plays an important role in regulating ischemic myocardial oxidative stress. Furthermore, the overexpression of miR-200a was found to protect cardiomyocytes from hypoxia-induced cell damage and the excessive production of reactive oxygen species [27]. On the other hand, the *G* allele of the c.*773 A/G polymorphism produced a binding site for miR-638; this microRNA plays an important role in the vascular smooth muscle cell (VSMC) proliferation and migration in atherosclerotic plaque vulnerability through the regulation of the cyclin D and NOR1. Alternatively, miR-638 is a regulator of the platelet-derived growth factor-BB (PDGF-BB), which is released primarily by vascular endothelial cells and platelets at the sites of vascular injury. Of note, this miRNA has been identified as one of the most potent stimulants for the VSMC proliferation and migration, through the modulation of several transcription factors and key molecular signaling pathways [28,29]. However, the effects of miR-200a or miR-638 on plasma lipid levels has not been previously reported. Mechanistically, using bioinformatics tools, miRNAs predicted to recognize the polymorphic site were expected to decrease the LDL-R-mRNA half-life in the cytoplasm [19,20]. Consequently, LDL-cholesterol should have been higher in the risk allele carriers, but such difference was not observed. Speculative explanations to these observations include a mRNA stabilizing role of the micro RNAs or a limited or null association of the miRNA to the 3′UTR region. Based in the LDL-cholesterol plasma levels it is likely that the second result is more acceptable; the miR-200a and miR-638 seem to have a little impact on regulating LDL-R gene expression. Nevertheless, it cannot be discarded a contribution of these miRNAS to increasing LDL-cholesterol plasma levels. To our knowledge, there are no data describing the possible pathways altered specifically in ACS and/or other cardiovascular events by the miR-200a and miR-638, and binding sites harboring to the alleles c. * 52 A and c. * 773 G. Nonetheless, recent data have identified several SNPs that generates binding sites with microRNAs such as miR-33, miR-148a, and miR.128-1 that play an important role in the LDLR expression [30]. Future investigations are needed to understand the effect of these polymorphisms on LDL-R and HDL-C plasma levels and its potential relationship with miRNAs.

Finally, in our study, the c.*52 *A/G* and c.*773 *A/G* polymorphisms were associated with the presence of ACS; however, the participation of these polymorphisms is controversial in other populations. We think that the association of the *LDLR* polymorphisms with ACS could be due to the role of this receptor in the regulation of the circulating cholesterol-containing lipoprotein particles, which is, in turn, an important cardiovascular risk factor [11,23]. It is important to notice that the allele distribution of these polymorphisms varies according to the ethnic origin of the study populations. In this context, data obtained from the National Center for Biotechnology Information revealed that the individuals from Los Angeles with Mexican ancestry, Mexican mestizos, Caucasian, and Africans had a lower frequency of the c.*52 A allele (26, 23, 23, and 15%, respectively) than Asians (41%). Moreover, Mexican mestizos, Europeans, Africans, as well as individuals from Los Angeles with Mexican ancestry, have a lower frequency of the c.*773 G allele (23, 22, 16, and 26%, respectively) than the Asian population (41%) [31]. Of note, the Mexican population has a characteristic genetic background with important ethnic differences compared to other populations [32,33,34]. Therefore, we consider that studies with a greater sample in populations with different ethnic origins may explain the true role of *LDLR* SNPs in the risk of developing ACS.

In summary, this study demonstrated that the c.*52 A/G and c.*773 A/G polymorphisms of the *LDLR* gene are associated with the risk of developing ACS in a Mexican population. In addition, it was possible to distinguish one haplotype (*AAG*) associated with a higher risk of developing ACS. There was a statistically significant association of both c.*52 A/G and c.*773 A/G polymorphisms with lower HDL-C levels in plasma. Lastly, because of the specific genetic characteristics of the Mexican population, we consider that additional studies need to be undertaken in a larger number of individuals and in populations with different ethnic origins. This future research could help define the true role of these polymorphisms as markers of risk or protection from developing ACS and other cardiovascular events.

## Figures and Tables

**Table 1:** Anthropometrics and biochemical parameters of the study individuals.

Characteristic		ACS Patients (n = 618)	Healthy Controls (n = 666)	*p*-Value
		Median (percentile 25–75)	Median (percentile 25–75)	
Age (years)		58 (51–65)	54 (49–59)	0.001
Gender n (%)	Male	505 (82)	453 (68)	<0.001
	Female	113 (18)	213 (32)	
BMI (kg/m2)		27 (25–29)	28 (26–31)	0.521
Blood pressure (mmHg)	Systolic	132 (114–144)	117 (106–126)	<0.001
	Diastolic	80 (70–90)	73 (67–78)	<0.001
Glucose (mg/dL)		159 (102–188)	98 (84–99)	<0.001
Total cholesterol (mg/dL)		164(128–199)	191 (165–210)	<0.001
HDL-C (mg/dL)		39 (34–45)	44 (35–53)	0.017
LDL-C (mg/dL)		106 (75–132)	116 (94–134)	<0.001
Triglycerides (mg/dL)		169 (109–201)	176 (113–208)	0.301
Hypertension n (%)	Yes	350 (57)	201 (30)	<0.001
Type II diabetes mellitus n (%)	Yes	216 (35)	63 (9)	<0.001
Dyslipidemia n (%)	Yes	528 (85)	479 (72)	<0.001
Smoking n (%)	Yes	222 (36)	147 (22)	<0.001

Data are expressed as median and percentiles (25th–75th). *p* values were estimated using the Mann-Whitney *U* test for continuous variables and the chi-squared test for categorical values. ACS: Acute coronary syndrome.

**Table 2:** Distribution of *LDLR* polymorphisms in ACS patients and healthy controls.

Polymorphic Site		n (Genotype Frequency)		Model	OR (95%CI)	*pC*	n (Allele Frequency)		*OR*	*95%CI*	*pC*
*LDLR UTR′3*	c.*52 *A/G* (rs14158)							*Risk allele			
Control	*GG*	*AG*	*AA*				*G*	*A	*A vs. G*		
(n = 666)	385 (0.578)	252 (0.378)	29 (0.043)	Co-dominant	2.02 (1.18–3.46)	0.033	1022 (0.766)	310 (0.232)			
				Dominant	1.13 (0.88–1.45)	0.35			1.20	1.00–1.44	0.02
ACS	337 (0.545)	231 (0.374)	50 (0.081)	Recessive	2.00 (1.18–3.39)	0.009	905 (0.732)	331 (0.267)			
(n = 618)				Over-dominant	0.96 (0.74–1.24)	0.73					
				Additive	1.20 (0.98–1.45)	0.075					
*LDLR UTR′3*	c.*504 *A/G* (rs2738465)										
Control	*GG*	*AG*	*AA*				*G*	*A	*A vs. G*		
(n = 666)	323 (0.485)	283 (0.425)	60 (0.090)	Co-dominant	1.50 (0.99–2.26)	0.16	929 (0.696)	403 (0.302)			
				Dominant	1.15 (0.90–1.47)	0.27			1.18	1.00–1.40	0.02
ACS	280 (0.453)	256 (0.414)	82 (0.133)	Recessive	1.45 (0.98–2.16)	0.06	816 (0.660)	420 (0.339)			
(n = 618)				Over-dominant	0.99 (0.77–1.27)	0.94					
				Additive	1.17 (0.97–1.41)	0.09					
*LDLR UTR′3*	c.*773 *A/G* (rs2738466)										
Control	*AA*	*AG*	*GG*				*A*	*G	*G vs. A*		
(n = 666)	386 (0.580)	250 (0.375)	30 (0.045)	Co-dominant	2.04 (1.35–3.45)	0.027	1022 (0.766)	310 (0.232)			
				Dominant	1.14 (0.88–1.46)	0.32			1.22	1.02–1.146	0.01
ACS	335 (0.542)	231 (0.374)	52 (0.084)	Recessive	2.01 (1.20–3.38)	0.007	901 (0.728)	335 (0.271)			
(n = 618)				Over-dominant	0.96 (0.74–1.24)	0.73					
				Additive	1.21 (0.99–1.48)	0.062					

ACS, Acute coronary syndrome; OR, odds ratio; CI, confidence interval; *p*C, *p*-value. The *p*-values were calculated with the logistic regression analysis, and ORs were adjusted for gender, age, blood pressure, BMI, glucose, total cholesterol, HDL-C, LDL-C, triglycerides, and smoking habit.

**Table 3:** Frequencies of *LDLR* haplotypes in patients with ACS and healthy controls.

c.*52 A/G	c.*504 A/G	c.*773 A/G	ACS (n = 618)	Controls (n = 666)	OR	95%CI	*pC*
Haplotype			Hf	Hf			
*G*	*G*	*A*	0.658	0.695	0.84	0.71–0.99	0.023
*A*	*A*	*G*	0.267	0.230	1.22	1.02–1.46	0.016
*A*	*G*	*A*	0.070	0.069	1.03	0.76–1.39	0.446

Abbreviations: ACS: acute coronary syndrome; Hf = Haplotype frequency, *pC* = *p* corrected. The order of the polymorphisms in the haplotypes is according to the positions in the chromosome (rs14158, rs2738465, rs2738466).

## Supplementary Materials

The following are available online at https://www.mdpi.com/2218-273X/10/10/1381/s1, Table S1: Association of the LDLR gene SNPs with plasma lipids levels and anthropometric characteristics in the healthy control group (n = 666).

## Abbreviations

LDLR	Low-density lipoprotein receptor
UTR′3	Untranslated region-′3
HDL-C	High-density lipoprotein-cholesterol
LDL-C	Low-density lipoprotein-cholesterol
T2DM	Type 2 diabetes mellitus
SNP	Single nucleotide polymorphism
ACS	Acute coronary syndrome
