# Supplementary material for: The c.*52 A/G and c.*773 A/G Genetic Variants in the UTR′3 of the LDLR Gene Are Associated with the Risk of Acute Coronary Syndrome and Lower Plasma HDL-Cholesterol Concentration
Source: Biomolecules. 2020 Sep 29;10(10):1381. doi: 10.3390/biom10101381 (PMC7599626; doi:10.3390/biom10101381)
Supplement: Supplementary file 1 [file biomolecules-10-01381-s001.pdf]

Table 1. Association of the *LDLR* gene SNPs with plasma lipids levels and anthropometric characteristics in the healthy control group (n = 666).

LDLR	***c.*773 A/G***			***p-value***	***p-value***
	AA (n *= 386)*	*AG* (n *= 250)*	*GG* (n *= 30)*	*AA vs GG**	*AG vs GG***
Parameters					
BMI (kg/m^2)	28.4 ± 4.0	28.2 ± 4.1	27.6 ± 3.6	0.264	0.415
Blood pressure (mmHg)					
Systolic	118 ± 16.7	117 ± 15.6	114.2 ± 11.0	0.305	0.296
Diastolic	73 ± 9.3	72 ± 8.4	71 ± 7.0	0.336	0.71
Glucose (mg/dL)	97.1 ± 26.5	101 ± 38.8	97.3 ± 24.0	0.79	0.709
Total cholesterol (mg/dL)	191.6 ± 38.6	190 ± 36.3	185.9 ± 38.9	0.383	0.424
HDL-C (mg/dL)	44.4 ± 13.1	45.4 ± 14.9	38.2 ± 10.3	0.007*	**0.003***
LDL-C (mg/dL)	117.6 ± 33.1	114.1 ± 30.8	111.8 ± 23.6	0.312	0.588
Triglycerides (mg/dL)	173 ± 89.0	178.9 ± 103.3	190.9 ± 108.6	0.631	0.656

LDLR	***c.*504 A/G***			***p-value***	***p-value***
	GG (n *= 323)*	*GA* (n *= 283)*	*AA* (n *= 60)*	*GG vs AA**	*GA vs AA**
Parameters					
BMI (kg/m^2)	28.5 ± 4.1	28.1 ± 4.0	28.2 ± 4.2	0.653	0.948
Blood pressure (mmHg)					
Systolic	118.2 ± 17.0	116.6 ± 15.4	117.6 ± 13.7	0.955	0.589
Diastolic	73.2 ± 9.2	71.7 ± 8.6	73.4 ± 7.8	0.729	0.092
Glucose (mg/dL)	97.6 ± 27.4	99.5 ± 35.4	98.9 ± 34.0	0.771	0.523
Total cholesterol (mg/dL)	191.7 ± 39.3	190.5 ± 37.1	187.9 ± 32.4	0.655	0.767
HDL-C (mg/dL)	44.6 ± 13.4	44.9 ± 13.3	41.7 ± 13.8	0.060	0.039*
LDL-C (mg/dL)	117.5 ± 33.0	115.1 ± 32.3	112.1 ± 22.2	0.259	0.505
Triglycerides (mg/dL)	171 ± 87.9	177.4 ± 98.7	195.8 ± 116.0	0.285	0.384

LDLR	***c.*52 A/G***			***p-value***	***p-value***
	GG (n *= 385)*	*GA* (n *= 252)*	*AA* (n *= 29)*	*GG vs AA**	*GA vs AA**
Parameters					
BMI (kg/m^2)	28.4 ± 4.0	28.2 ± 4.1	27.6 ± 3.6	0.329	0.455
Blood pressure (mmHg)					
Systolic	117.9 ± 16.6	117.1 ± 15.6	114.3 ± 11.1	0.349	0.331
Diastolic	73.0 ± 9.3	72.1 ± 8.4	71.6 ± 7.1	0.389	0.78
Glucose (mg/dL)	97.5 ± 28.2	100.4 ± 36.8	97.3 ± 24.4	0.862	0.873
Total cholesterol (mg/dL)	191.9 ± 38.7	190 ± 36.1	184.1 ± 38.3	0.239	0.304
HDL-C (mg/dL)	44.3 ± 13.2	45.5 ± 13.7	38.0 ± 10.4	0.007*	0.002**
LDL-C (mg/dL)	117.8 ± 33.2	114.1 ± 30.7	109.7 ± 20.7	0.180	0.411
Triglycerides (mg/dL)	173.7 ± 89.5	177.5 ± 102.5	192.8 ± 110.1	0.604	0.584

Abbreviations: BMI = Body mass index, HDL = High-density lipoprotein – cholesterol, LDL: Low density lipoprotein, *p* = *p-value.* Data of BMI, blood pressure, glucose, total cholesterol, HDL-C, LDL-C, and triglycerides and Ratio LDL-C/HDL-C are expressed as mean ± SD adjusted by gender and age.
